# Pan-cancer analysis of kinesin family members with potential implications in prognosis and immunological role in human cancer

**DOI:** 10.3389/fonc.2023.1179897

**Published:** 2023-08-29

**Authors:** Ming Zhong, Lian Gong, Na Li, Hui Guan, Kai Gong, Yong Zhong, Enyi Zhu, Xiaohua Wang, Shan Jiang, Jinhong Li, Yan Lei, Yu Liu, Jiasi Chen, Zhihua Zheng

**Affiliations:** ^1^Department of Nephrology, Center of Kidney and Urology, The Seventh Affiliated Hospital, Sun Yat-sen University, Shenzhen, China; ^2^Department of Oncology, The Seventh Affiliated Hospital, Sun Yat-sen University, Shenzhen, China; ^3^Department of Oncology, Third Xiangya Hospital, Central South University, Changsha, China; ^4^Edmond H. Fischer Translational Medical Research Laboratory, Scientific Research Center, The Seventh Affiliated Hospital, Sun Yat-Sen University, Shenzhen, China; ^5^Division of Hepatobiliary and Pancreatic Surgery, Department of General Surgery, The Second Affiliated Hospital of Dalian Medical University, Liaoning, China; ^6^Department of Clinical Medicine, Hubei Enshi College, Enshi, China

**Keywords:** kinesin family member, pan-cancer, immune, machine learning, genomic variation

## Abstract

**Background:**

Kinesin is a molecular motor for transporting “goods” within cells and plays a key role in many types of tumors. The multi-angle study of kinesin at the pan-cancer level is conducive to understanding its role in tumorigenesis and development and clinical treatment potential.

**Methods:**

We evaluated the expression of KIF genes, performed differential analysis by using the R package limma, and explored the pan-cancer prognosis of KIF genes by univariate Cox regression analysis. To evaluate the pan-cancer role of KIF genes as a whole, we defined the KIFscore with the help of gene set variation analysis (GSVA) and explored the KIFscores across normal tissues, tumor cell lines, and 33 tumor types in TCGA. Next, we used spearman correlation analysis to extensively study the correlation between the KIFscore and tumor prognosis and be-tween the KIFscore and clinical indicators. We also identified the relationship between the KIFscore and genomic variation and immune molecular signatures by multiplatform analysis. Finally, we identified the key genes in clear cell renal cell carcinoma (ccRCC) through machine learning algorithms and verified the candidate genes by CCK8, wound healing assay, Transwell assay, and flow cytometry.

**Results:**

In most cancers, KIFscores are high and they act as a risk factor for cancer. The KIFscore was significantly associated with copy number variation (CNV), tumor mutation burden (TMB), immune subtypes, DNA repair deficiency, and tumor stemness indexes. Moreover, in almost all cancer species, the KIFscore was positively correlated with T cell CD4+ TH2, the common lymphoid pro-genitor, and the T cell follicular helper. In addition, it was negatively correlated with CXCL16, CCL14, TNFSF13, and TNFRSF14 and positively correlated with ULBP1, MICB, and CD276. Machine learning helped us to identify four hub-genes in ccRCC. The suitable gene, KIF14, is highly expressed in ccRCC and promotes tumor cell proliferation, migration, and invasion.

**Conclusion:**

Our study shows that the KIF genes play an important pan-cancer role and may become a potential new target for a variety of tumor treatments in the future. Furthermore, KIF14, a key molecule in the KIF genes, can provide a new idea for the ccRCC treatment.

## 1 Introduction

The incidence of and mortality rate from cancer continue to increase, with cancer becoming the second leading cause of death in the world, seriously threatening human health and imposing huge health and economic burden on society (1). Cancer is a disease with multi-omic dysregulation, and the most basic characteristics of cancer cells include continuous proliferation and mitosis by promoting the production and release of growth signals that affect cancer growth and progression (2). Therefore, targeting this biological process is expected to prevent the occurrence and progression of cancer.

The kinesin superfamily proteins (KIFs) are important molecular motors for the intracellular transport of “goods.” The main function of KIFs is chromosome aggregation, spindle formation, and intracellular material transport during cell mitosis (3, 4). KIFs are mainly divided into 14 kinesin subfamilies (Kinesin1~Kinesin14), with a total of 45 kinesin family members (5). Kinesin abnormalities can change the distribution of genetic material in cells through chromosomal overagglutination, spindle formation abnormalities, cell division defects, late bridge formation or aneuploidy, and mitosis blockade; result in an out-of-control cell cycle; and play an important role in the development of malignant tumors and drug resistance. For example, KIF3A and KIF13A are essential for cancer cell migration (6), a Kinesin13 family member MCAK leads to tumor invasion and paclitaxel resistance (7, 8), and KIF20A and KIF15 contribute to the castration and Enzalutamide resistance of prostate cancer (9, 10). It can be seen from the above that KIFs are key molecules in a variety of human cancers. Therefore, a systematic study of KIFs in cancer can help better understand their role and clinical therapeutic potential in cancer. However, there are a large number of kinesins, most of the previous studies are based on the single-tumor-type exploration by a single kinesin molecule, and there is no research that comprehensively evaluates the role of KIFs in tumors. In addition, different kinesins perform different functions in tumorigenesis and development and the identification of key carcinogenic or cancer-suppressing kinesins can facilitate follow-up research to further explore the pathogenesis of tumors and provide a new target for tumor treatment.

Given the above, we analyzed the expression and prognosis of KIFs to evaluate their role at the pan-cancer level and constructed KIFscores by GSVA to quantify KIFs and evaluate the overall pan-cancer role of KIFs. Next, we further explored the KIFscore levels of normal tissues, tumor cell lines, and patients at the pan-cancer level through various bioinformatics methods and studied in depth the correlation between the KIFscore and tumor prognosis, clinical indicators, genomic variations, and immune molecular characteristics so as to identify the role of KIFs in tumorigenesis and development. Finally, we screened the key KIFs in ccRCC through machine learning and verified their role in ccRCC using a variety of experimental methods.

## 2 Materials and methods

### 2.1 Data and resources

Gene expression data for 33 cancer types containing 11,066 samples and clinical information about patients were downloaded from the TCGA database (https://gdc.cancer.gov/about-data/publications/pancanatlas). The R package TCGAbiolinks (version 2.24.3) was used to download Somatic mutation data (11), including copy number variations (CNVs) and single-nucleotide variants (SNVs). We obtained gene expression profiles from the Genotype-Tissue Expression Project (GTEx) database (https://www.gtexportal.org/home/datasets) and cancer cell line gene expression profiles from the Cancer Cell Line Encyclopedia (CCLE) database (https://sites.broadinstitute.org/ccle/). We downloaded the stemness score (RNAss and DNAss) and information about homologous recombination deficiency (HRD), homologous recombination deficiency–loss of heterozygosity (HRD–LOH), and immune subtypes of TCGA samples from the UCSC Xena database (https://xenabrowser.net/datapages/). In all, data on 45 KIF genes were collected from the published literature, of which only 38 KIF genes were present in the expression matrix we adopted (**Supplementary Table 1**).

### 2.2 KIFscore calculation

We performed univariate Cox regression analysis on 38 KIF genes present in all tumors on which overall survival data (OS) was available (LAML lacks OS data) and found the low-risk gene (p < 0.05; HR < 1) and the high-risk gene (p < 0.05; HR > 1) in each tumor. When the number of high-risk genes in all tumors is greater than the number of low-risk genes, it is defined as a positive gene. Otherwise, it is a negative gene. Next, we used the GSVA algorithm of the R package GSVA to calculate the activities of two sets for each sample (12, 13). The activity score of the positive gene minus the activity score of the negative gene is the KIFscore. Tumor samples were divided into high- and low-KIFscore groups by the median KIFscore.

### 2.3 Landscape of the KIFscore at the pan-cancer level

Using the above KIFscore calculation method, we calculated the KIFscores for normal tissue from the Genotype-Tissue Expression Project, cancer cell lines from the Cancer Cell Line Encyclopedia, and tumor samples from the Cancer Genome Atlas database. Finally, we obtained the average KIFscores of each normal tissue, cancer cell line, and tumor type to assess the KIFscore levels. Meanwhile, KIFscores of paired tumor samples from TCGA were compared to explore the differences in KIFscores between cancerous and adjacent tissue.

### 2.4 Prognostic analysis of the KIFscore

We performed Cox regression analysis of the KIFscore on various survival indicators, including overall survival (OS), disease-specific survival (DSS), disease-free interval (DFI), and progression-free interval (PFI). The cancer types with favorable survival (p < 0.05; HR < 1) were considered cancer types with protective KIFscore-related survival, while cancer types with poor survival (p < 0.05; HR > 1) were considered cancer types with risk KIFscore-related survival. Kaplan–Meier analysis was used to further explore survival among patients with high and low KIFscores (14). We conducted more in-depth validation of individual tumor types, such as clear cell renal cell carcinoma, and used Fisher test to explore and compare various clinical indicators of clear cell renal cell carcinoma in the high- and low-KIFscore groups.

### 2.5 Genomic variation analysis

To identify arm-level and focal-level event changes, we downloaded the masked copy number segments of 23 tumor types with a sample size greater than 100 and analyzed them by GISTIC 2.0 (15). It was considered an important widespread event when more than 70% of the arms had undergone mutation with q values < 0.25. Subsequently, we defined CNV scores based on previous research (16). For focal-level events, the scores were divided by the ratios of log2 copy number as follows: 2 if the log2 ratio ≥ 1, 1 if the log2 ratio < 1 and ≥0.25, 0 if the log2 ratio < 0.25 and ≥−0.25, −1 if the log2 ratio < −0.25 and ≥−1, and −2 if the log2 ratio < −1. To obtain the focal score of a tumor, all focal-level scores were added. A similar procedure was used to define the arm- and chromosome-level scores. When both arms had the same log2 ratio, it was considered a chromosome-level event. The focal-level, arm-level, and chromosome-level CNV scores added up to the overall CNV score of the tumor. Subsequently, covariates such as age, gender, and race were adjusted and a linear model was applied to determine the association between the KIFscore and the CNV score. When log2-transformed copy number ratios are >0.25 and <−0.25, arm-levels are defined as arm-level gains and losses, respectively. Using a linear model similar to the one above, we calculated the correlation between the KIFscore and arm-level gains and losses. We also explored the correlation between the KIFscore and the TMB using the above approach. The R package maftools was used to plot a mutant waterfall chart of ccRCC patients with high and low KIFscores (17).

### 2.6 Immune characteristics of the KIFscore at the pan-cancer level

We downloaded the immune scores of all patients from the TIMER 2.0 database (http://timer.cistrome.org/). The R package ESTIMATE was used to assess the tumor immune microenvironment, such as the stromal score, the immune score, the ESTIMATE score, and tumor purity. Information about immunomodulators, the major histocompatibility complexes (MHCs), chemokines, and chemokine receptors were collected from published literature (**Supplementary Table 2**). The correlation between the above data and the KIFscore was calculated using spearman correlation analysis. Next, we obtained the immune activity scores of all patients with different tumors from the Tracking Tumor Immunophenotype database (http://biocc.hrbmu.edu.cn/TIP/). The Wilcoxon test was used to compare the differences in the anti-cancer immune statuses of patients with high and low KIFscores.

### 2.7 Identification of key KIF genes in ccRCC

We performed differential analysis (|log2FC| > 1; padj < 0.05) on ccRCC samples grouped by high and low KIFscores. We used the Least Absolute Shrinkage and Selection Operator (LASSO) logistic regression, analyzed by the R package glmnet (18), Random Forest (RF) (analyzed by R package randomForest), Support Vector Machine–Recursive Feature Elimination (SVM–RFE) (19, 20), to screen candidate genes. The overlapping genes of the three algorithms are considered key genes for ccRCC.

### 2.8 Cell culture and siRNA delivery

We purchased the renal clear cell carcinoma cell line 786-O and 769-P, as well as the renal tubular epithelial cell line HK2, from the Chinese Academy of Science (Shanghai, China), and cultured the cells in a 37°C incubator with 1640 medium supplemented with 10% fetal bovine serum and 1% penicillin/streptomycin solution (21). We used siKIF14s to knock down KIF14, which we had purchased from GenePharma (Shanghai, China). The siRNA sequences were as follows: siKIF14#1, sense 5’-CAGCGGUGAUAUUCUUGAUTT-3’sense and antisense 5’-AUCAAGAAUAUCACCGCUGTT-3’; siKIF14#2, sense 5’-GCCCGUUUAAUAGUCAACATT-3’ and antisense 5’-UGUUGACUAUUAAACGGGCTT-3’; negative control (NC), sense 5’-UUCUCCGAACGUGUCACGUTT-3’ and antisense 5’-ACGUGACACGUUCGGAGAATT-3’. We collected cells for RT-qPCR, proliferation, and migration experiments 24 h after siRNA and lipo2000 transfection.

### 2.9 RNA isolation and quantitative real−time PCR

To extract the total RNA, we used RNA fast200 (Fastagen, Shanghai, China). The qRT−PCR primers were as follows: GAPDH, forward 5’-GTCAGCCGCATCTTCTTT-3’ and reverse 5’-CGCCCAATACGACCAAAT-3’; KIF14, forward 5’-GCACTTTCGGAACAAGCAAACCA-3’ and reverse 5’-ATGTTGCTGGCAGCGGGACTAA-3’. To quantify the gene expression level, we used the 2−ΔΔCt methods.

### 2.10 CCK-8 assay

We seeded the cells in 96-well plates at a density of 2 × 10^3^ cells per well and detected cell viability after 24 h for 4 consecutive days (D0, D1, D2, and D3). We discarded the culture medium, mixed CCK-8 (Biosharp, Anhui, China) reagent with the culture medium in the ratio of 1:10, and added 110 μl of mixed solution to each well. After the mixture was incubated at 37°C for 1.5 h, the OD value at 450 wavelength was detected on the microplate. Next, we set the wavelength of the microplate reader to 450 nm and detected the OD value.

### 2.11 Wound healing assay

We seeded cells in 6-well plates at a density of 2.5 × 10^5^ cells per well and transfected the cells with siKIF14#1, siKIF14#2, or siNC. After the cells covered the 6-well plate, we used a 200 μL pipette tip to scrape the cells, washed the cells with PBS, and replaced the serum-free medium. Finally, we took images at 0 h and 24 h under a microscope.

### 2.12 Transwell assay

We inoculated 1×10 4 cells in the upper chamber with 200 μl of 1640 medium and added 600 μL of 1640 medium containing 20% FBS to the lower chamber. After 24 h, we stained the cells with crystal violet, wiped off the cells in the upper chamber, and took photos with a microscope. We used ImageJ to count the number of cells passing through the upper chamber in four random fields under the microscope.

### 2.13 Flow cytometry assay

After transfection of 786-O and 769-P cells in a 6-well plate, we collected the cells in a culture medium, centrifuged the cells, and washed the cells with PBS. We added Annexin V-FITC conjugate solution to resuspend the cells, added Annexin V-FITC and propidium iodide staining solution, mixed the mixture well, and incubated the mixture at room temperature, protected from light, for 15 min. We tested with flow cytometry and analyzed the results with FlowJo.

### 2.14 Statistical analysis

To compare the two groups, we used unpaired t-test and Wilcoxon rank-sum test. To analyze the differences among multiple groups, we used the Kruskal–Wallis test. To analyze the correlation between variables, we used linear model and the spearman correlation test. Continuous normally distributed variables were shown as mean ± standard deviation and compared using one-way ANOVA. R software (version 4.0.3), GraphPad Prism (version 9.4.1), and Adobe Illustrator (version 25.0) were used for statistical analysis and drawing. We considered p < 0.05 as statistically significant.

## 3 Results

### 3.1 KIF gene expression and prognosis analysis

**Figure 1:**
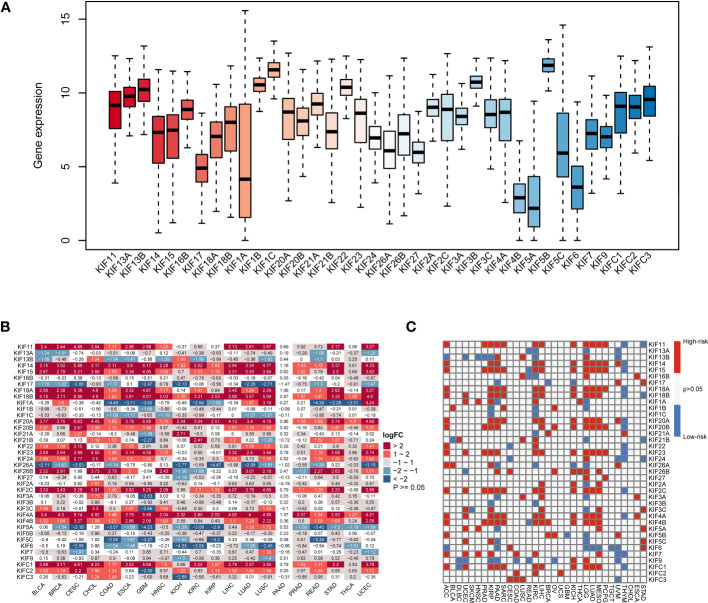
The expression of KIF genes in normal and tumor tissues. **(A)** The expression levels of the KIF genes in 33 tumor types. **(B)** Differential expression analysis of KIF genes in 20 tumor types. Red sections represent upregulated expression of KIF genes in tumors, and blue sections represent downregulated expression of KIF genes in tumors. **(C)** Univariate Cox regression analysis of KIF genes at the pan-cancer level, and the definition of high-risk and low-risk genes. Red parts represent the high-risk genes, and blue parts represent the low-risk genes.

The flowchart in **Supplementary Figure 1** presents the workflow of this study. First, we evaluated the expression of KIF genes in 33 tumor types (**Figure 1A**), of which the 5 genes with the highest expression were KIF5B, KIF1C, KIF1B, KIF3B, and KIF22, while the 5 genes with the lowest expression were KIF5A, KIF4B, KIF6, KIF1A, and KIF17. Next, we carried out differential analysis of KIF genes in 20 tumor types containing both tumor and normal samples (**Figure 1B**). The results showed that KIF11, KIF14, KIF15, KIF18A, KIF18B, KIF20A, KIF23, KIF2C, KIF4A, and KIFC1 were highly expressed in most tumors, while KIF17, KIF26A, KIF5A, and KIF6 were expressed at low levels in most tumors. After pooling clinical information, we performed the univariate Cox regression analysis of 38 KIF genes on eligible tumors (**Figure 1C**). As per the results, KIF11, KIF14, KIF15, KIF18A, KIF18B, KIF20A, KIF20B, KIF23, KIF26B, KIF2C, KIF4A, KIF4B, and KIFC1 were high-risk genes in the vast majority of tumors (p < 0.05; HR > 1) and KIF3B and KIF9 were mostly low-risk genes (p < 0.05; HR < 1).

### 3.2 The pan-cancer KIFscore landscape

**Figure 2:**
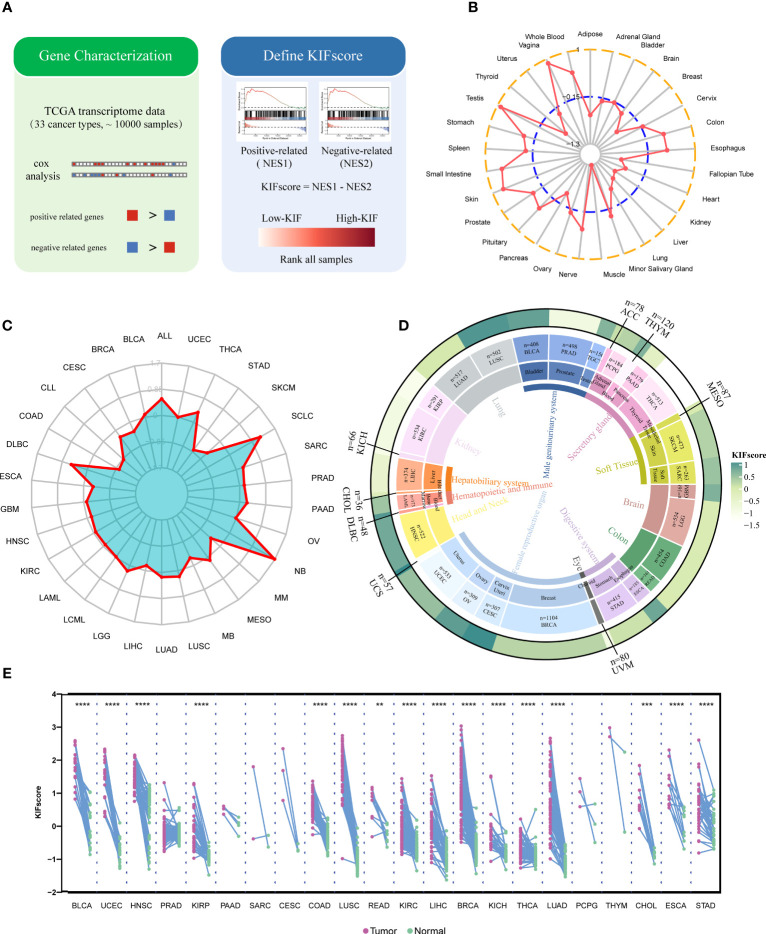
Comprehensive evaluation of KIF genes in normal tissues, tumor cell lines, and cancer patient samples. **(A)** The construction process of the KIFscore. **(B, C)** Average KIFscore across normal tissues **(B)** and CCLE cell lines **(C)**. **(D)** Average KIFscore in individual cancer types. Tissue types, cancer types, and average KIFscore are shown from the inner circle to the outer circle. **(E)** KIFscore level of paired tumor samples in TCGA. Purple stands for primary tumor, and green stands for paracancerous tissue (****P < 0.0001, ****P < 0.001, and **P < 0.01.

As per the above results, most KIF genes were differentially expressed in cancers and significantly affect tumor development and prognosis. However, there was no calculated measure to evaluate the overall level of KIF genes, so we quantified KIF genes by combining the prognostic effects of KIF genes on tumors to construct KIFscores (**Figure 2A**). A higher KIFscore means a greater gap between positive and negative KIF genes. Next, we analyzed the KIFscores in normal tissue (**Figure 2B**), the Cancer Cell Line Encyclopedia (CCLE) cell line (**Figure 2C**), and 33 cancers from TCGA (**Figure 2D**). In normal tissue, the tissues with the highest KIFscores were those from the testes, vagina, and the skin, while the tissues with the lowest KIFscores were those from the muscles, kidneys, and fallopian tubes. The three highest-KIFscore cell lines were NB, SCLC, and ALL, while the three lowest-KIFscore cell lines were COAD, CLL, and STAD. For the 33 tumor types of TCGA, we firstly classified organ system attribution and then applied the same method to evaluate KIFscore levels. The KIFscores of UCS and CESC derived from female reproductive organs, LUSC derived from lung tissue, ESCA derived from the digestive system were relatively high. Meanwhile, the KIFscores of THCA derived from the secretory system and KIRC derived from kidney tissue were relatively low (**Supplementary Table 3**). These results show that the KIFscore has a certain organizational preference. Subsequently, we analyzed the KIFscores in 20 paired tumors (**Figure 2E**). The results revealed that in many tumor types, the KIFscores in malignant tumors were higher than those in adjacent tissues, suggesting that tumor growth requires overexpression of KIFs. Overall, the results suggest that our method could vigorously quantify KIF genes levels and systematically describe the KIFscore landscape across 33 cancer types and 29 tissues.

### 3.3 Prognostic risk assessment of the KIFscore

**Figure 3:**
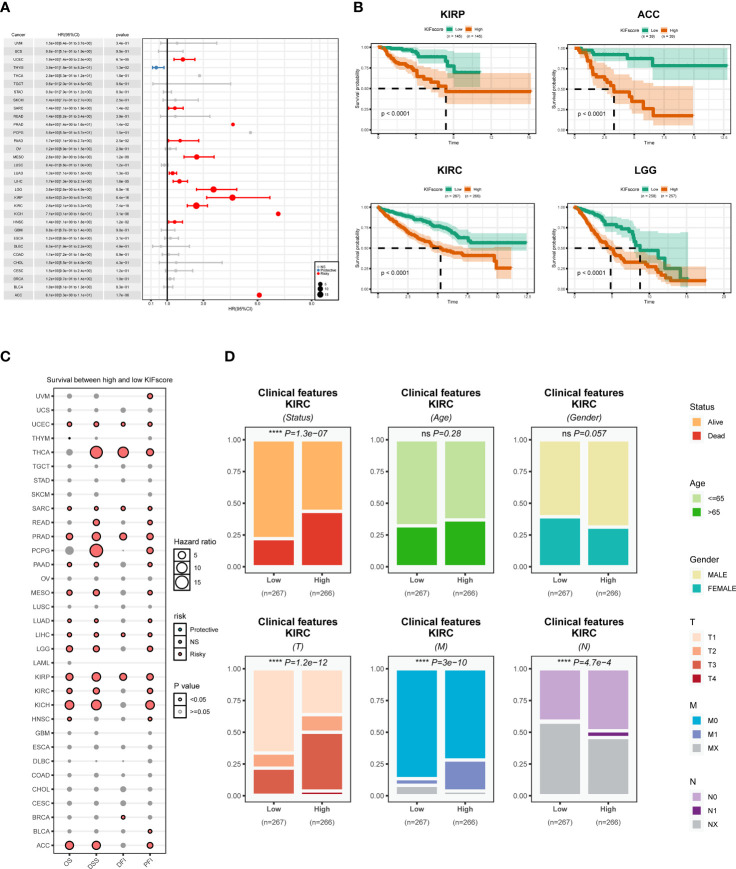
Prognostic analysis of the KIFscore at the pan-cancer level. **(A)** The forest plot shows the relationship between the KIFscore and the prognosis of each tumor. Red represents the risk factor in tumors, while blue represents the protective factor. **(B)** The survival curve plots show the relationship between the KIFscore and the prognosis of the four tumor types: ACC, KIRC, KIRP, and LG. **(C)** The bubble chart shows the relationship between the KIFscore and OS, DSS, DFI, and PFI of all tumors. **(D)** The percentage histogram reveals the differences in clinical indicators in the high-and low-KIFscore groups (****P < 0.0001 and ns P > 0.05).

To explore the ability of the KIFscore to determine the tumor prognostic risk, we performed univariate Cox regression analysis and found that the KIFscore was the risk factor (p < 0.05; HR > 1) in UCEC, SARC, PRAD, PAAD, MESO, LUAD, LIHC, LGG, KIRP, KIRC, HNSC, and ACC. The higher the KIFscore, the worse the prognosis for patients. As for THYM, the KIFscore was a protective factor (p < 0.05; HR < 1) and the lower the KIFscore, the better the prognosis for patients (**Figure 3A**). Next, we performed Kaplan–Meier analysis, which revealed that patients in the high-KIFscore group had a worse prognosis (**Figure 3B**; **Supplemental Figure 2**). As per the results of pan-cancer survival analysis (**Figure 3C**), the KIFscore was mostly a risk factor in malignant tumors. As per the analysis results, the KIFscore was a risk factor for 13 cancers in OS analysis, 15 cancers in DSS analysis, 7 cancers in DFI analysis, and 18 cancers in PFI analysis. On the basis of the previous differential expression analysis and prognosis analysis, it was suggested that patients with high KIFscores had a worse prognosis in ccRCC. Therefore, further clinical analysis of the KIFscore in ccRCC was carried out. The result showed that the KIFscore level had an effect on the alive status, the T stage, the N stage, and the M stage of ccRCC. The patients in the high-KIFscore group had high mortality rates and high stages of T, M, and N (**Figure 3D**).

### 3.4 Correlation analysis between the KIFscore and tumor genomic mutations

**Figure 4:**
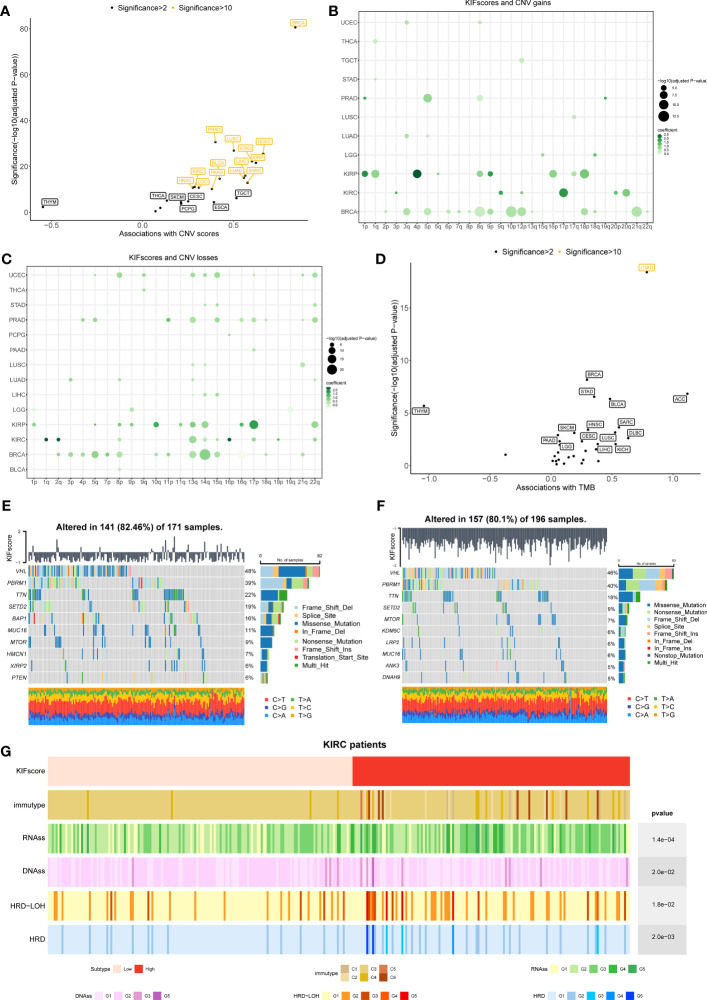
Correlation between the KIFscore and genomic variations was analyzed at the pan-cancer level. **(A)** Correlation between the KIFscore and the CNV score in different TCGA cancer types. The correlation and significance [−log10 (Benjamini–Hochberg-adjusted P-values)] in the linear model are shown on the x-axis and the y-axis, respectively. Tumors with significance greater than 2 are flagged, and tumors greater with significance greater than 10 are marked yellow. **(B, C)** Dot plots show the association between the KIFscore and the arm-level CNV gains **(B)** and the arm-level CNV losses **(C)**. The figure shows data with significance greater than 3. The circle size represents the size of the significance, and the colors represent the coefficients of linear regression. **(D)** Correlation between the KIFscore and the TMB at the pan-cancer level. **(E, F)** The waterfall chart shows the top mutation events for ccRCC patients in the high- **(E)** and low-KIFscore groups **(F)**. The bar plots in the top panel represent the KIFscores of individual patients. The panel on the right presents a statistical graph of mutation events for each gene. Colors represent variant classifications. **(G)** Distribution of tumor immunotypes in the high- and low-KIFscore groups of ccRCC patients, as well as RNAss, DNAss, HRD, and HRD–LOH.

One of the common markers of cancer is increased genomic instability. However, how genomic changes vary with KIFscores in a variety of cancers remains to be clarified. Therefore, our study explored the association of the KIFscore with CNV and TMB. To obtain CNV scores, we calculated the total of focal, arm, and chromosome levels in GISTIC2.0. The KIFscore was positively correlated with CNV scores of 20 malignant tumors except THYM. The correlation was particularly significant (significance > 10) in UCEC, KIRP, STAD, SARC, LIHC, LUAD, LUSC, PRAD, BLCA, PAAD, KIRC, LGG, and HNSC (**Figure 4A**). Next, we evaluated the correlation between arm-level CNV gains and losses and KIFscores in cancers with enough samples (**Figures 4B, C**). As per the results, in KIRP, the KIFscore was related to additions on chromosomes 1p, 1q, 4p, 9p, and 18p and deletions on chromosomes 10q, 13q, 14q, and 17p. In KIRC, the KIFscore was associated with additions on 9q, 17p, and 20q and deletions on 1q, 2q, 13q, and 16p (**Supplementary Table 4**). For TMB, the KIFscore was positively correlated with most cancers (**Figure 4D**). Next, we further explored the role of the KIFscore in tumor mutations in ccRCC (**Figures 4E, F**). To identify the difference in driver mutations between the high- and low-KIFscore groups, we analyzed the top mutated oncogenic genes of the high- and low-KIFscore groups. The first 10 mutated genes in ccRCC patients with high KIFscores were VHL, PBRM1, TTN, SETD2, BAP1, MUC16, MTOR, HMCN1, XIRP2, and PTEN, and the first 10 mutated genes in ccRCC patients with low KIFscores were VHL, PBRM1, TTN, SETD2, MTOR, KDM5C, LRP2, MUC16, ANK3, and DNAH9. In the high-KIFscore group, the mutation rate of VHL was 48%, and the mutations were mainly missense_mutation (47% of all mutation types). In the low-KIFscore group, the mutation rate of VHL was 46% and the proportion of missense_mutation (28%) decreased significantly, while that of nonsense_mutation increased significantly (from 10% to 23% in the high- and low-KIFscore groups). In the high-KIFscore group, the proportion of PBRM1 mutations was 39%, involving mainly Frame Shift Del mutations (46%). In the low-KIFscore group, the proportion of PBRM1 mutations was 40% and the Frame Shift Del (33%) decreased, while the nonsense_mutation increased from 25% to 27% (**Supplementary Table 5**). We also analyzed the relationship between the KIFscore and immune subtypes, the tumor stemness index, and DNA repair defects (**Figure 4G**). The patients with high KIFscores had more immune subtypes with poor prognosis, such as C1, C4, and C6. As per our results, the KIFscore was also significantly associated with the tumor stemness index and DNA repair defects.

### 3.5 Immune evaluation of the KIFscore

**Figure 5:**
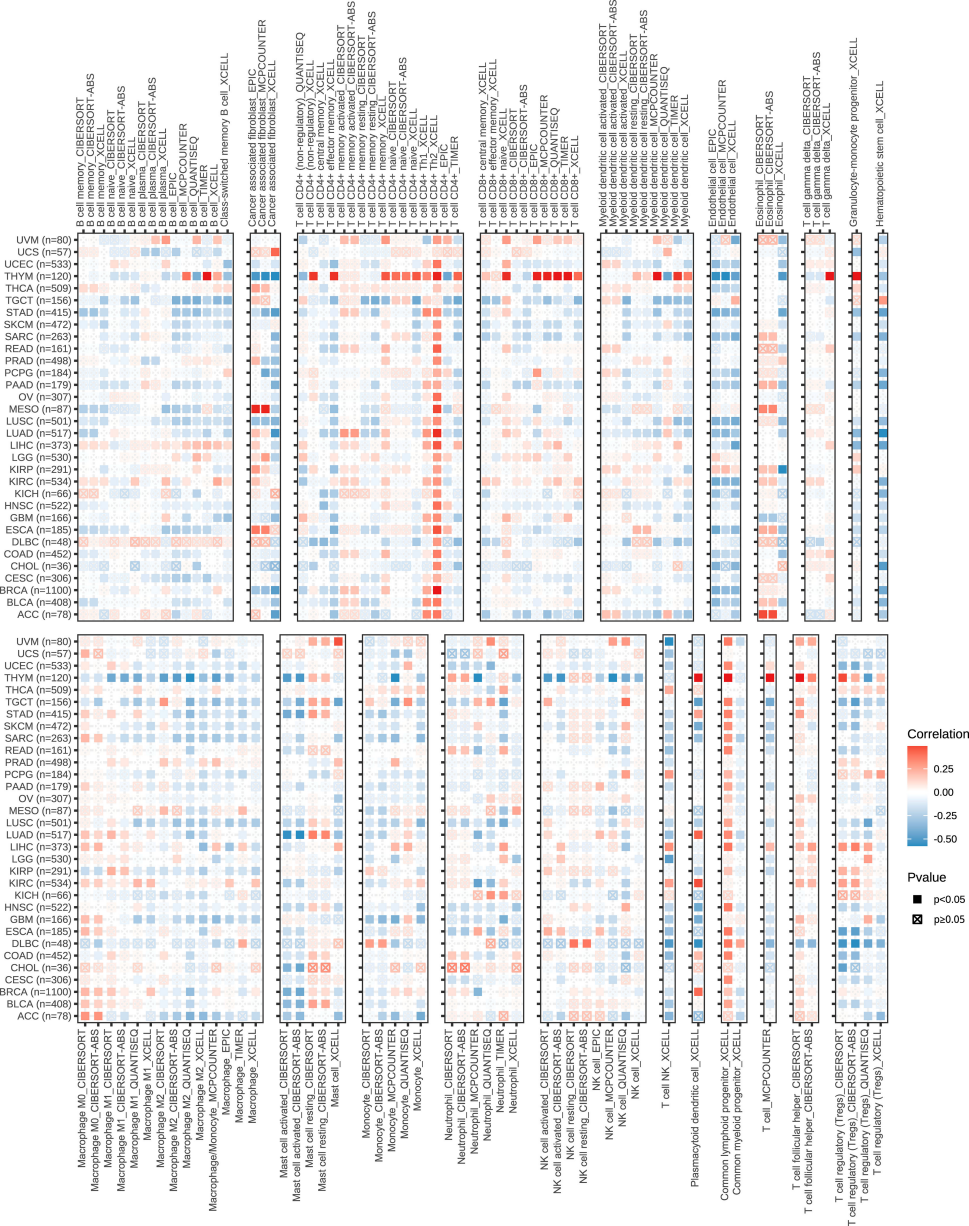
The correlation between the KIFscore and the infiltration levels of immune cells, such as B cells, cancer-associated fibroblasts, CD4+ T cells, CD8+ T cells, myeloid dendritic cells, Endo, Eos, T cell gamma delta, macrophages, mast cells, monocytes, neutrophils, NKT, and regulatory T cells (Tregs). Positive correlations are in red, and negative correlation are in blue.

**Figure 6:**
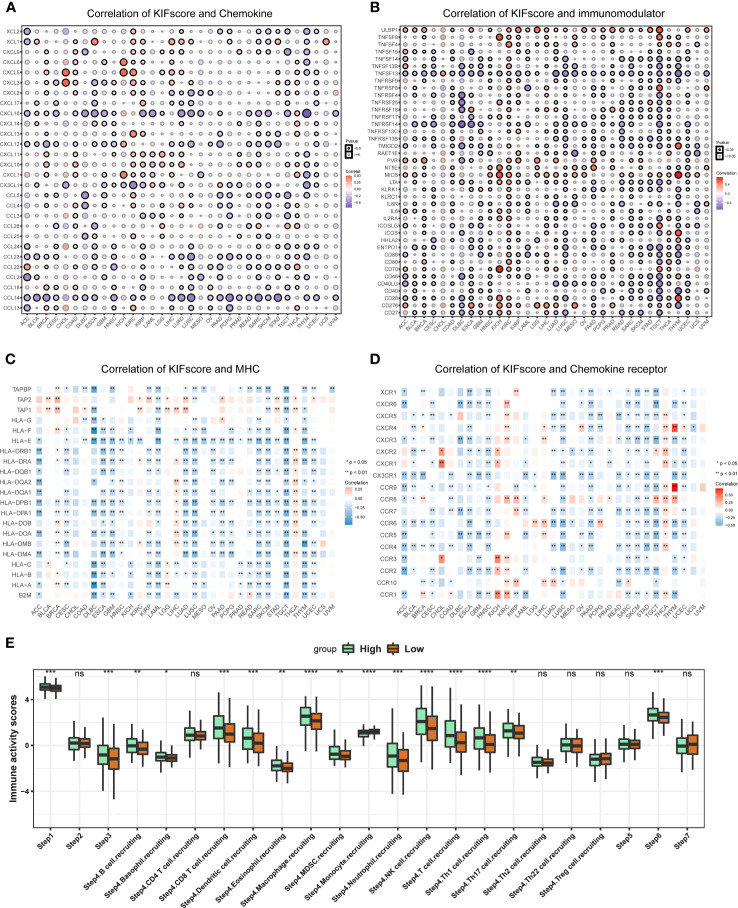
Immune correlation of KIFscores. **(A, B)** Correlation between the KIFscore and chemokines **(A)** and between the KIFscore and immunomodulars **(B)**. Positive correlations are in red, and negative correlations are in purple. **(C, D)** Correlation between the KIFscore and MHCs **(C)** and between the KIFscore and chemokine receptors **(D)**. Positive correlations are in red, and negative correlations are in blue. **(E)** The box plot shows the correlation between the KIFscore and the cancer-immunity cycle and between the KIFscore and immune cell infiltration in ccRCC. Green and orange represent high- and low-KIFscore groups, respectively (****P < 0.0001, ****P < 0.001, **P < 0.01, and *P < 0.05, respectively).

To explore the role of the KIFscore in tumor immune infiltration, we analyzed the correlation between the KIFscore and various immune cells in different tumors. In almost all cancers, the T cell CD4+ TH2, the common lymphoid progenitor, and the T cell follicular helper were found to be significantly positively correlated with KIFscores. In LIHC, THCA, KIRP, and KIRC, the KIFscore was positively correlated with Treg cells (**Figure 5**). We also analyzed the relationship between the KIFscore and immune-related genes. For chemokines (**Figure 6A**), our results suggest that the KIFscore was negatively correlated with CXCL16 and CCL14 in the vast majority of cancers. For immunomodulators (**Figure 6B**), we found that the KIFscore was positively correlated with ULBP1, MICB, and CD276, while it was negatively correlated with TNFSF13 and TNFRSF14 in the vast majority of cancers. In addition, in most tumors, the KIFscore was negatively correlated with most MHCs (**Figure 6C**) but positively correlated with TAP2 and TAP1. Furthermore, the KIFscore was positively correlated with most MHCs in THCA. The KIFscore was negatively correlated with the vast majority of chemokine receptors, but in KIRC and THCA, it was positively correlated with most chemokine receptors (**Figure 6D**). Next, we analyzed the correlation between the KIFscore and the cancer-immunity cycle and between the KIFscore and immune cell infiltration on the basis of the TIP database. The TIP database divides the cancer-immunity cycle into 7 steps: release of cancer antigens, cancer antigen presentation, priming and activation, trafficking of T cells to tumors, infiltration of T cells into tumors, recognition of cancer cells by T cells, and killing of cancer cells. As per our analysis, the KIFscore influenced steps 1, 3, 4, and 6 of ccRCC and the release of cancer antigens, priming and activation, trafficking of T cells to tumors, and recognition of cancer cells by T cells were more significant in the high-KIFscore group. In the high-KIFscore group, in step 4, B cell, basophil, CD8 T cell, dendritic cell, eosinophil, macrophage, MDSC, neutrophil, NK cell, T cell, Th1 cell, and Th17 cell recruitment were more pronounced. However, monocyte recruitment was inhibited (**Figure 6E**).

### 3.6 Characteristic gene screening

**Figure 7:**
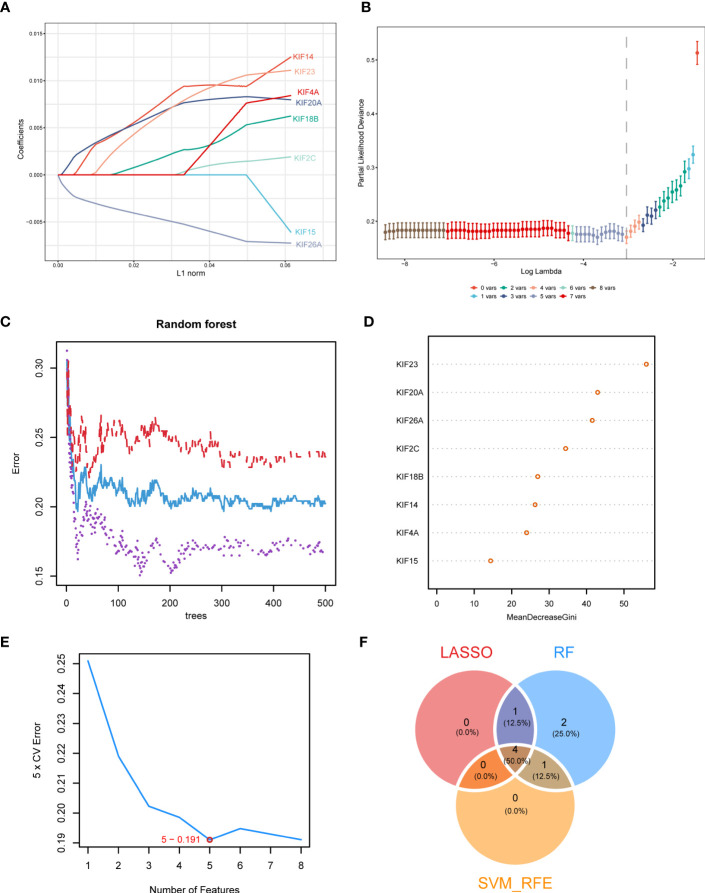
Identification of key genes in ccRCC. **(A, B)**. LASSO regression analysis was used to screen characteristic variables. **(C)** The relationship between the number of random forest trees and the error rate. Red, purple, and blue represented the high- KIFscore group, the low- KIFscore group, and the error of all samples, respectively. **(D)** Sequencing plot of genetic importance scores. **(E)** The error rate curve of the characteristic variable screening of the KIF genes using the SVM–RFE algorithm. The red circle is the point where the error rate was the lowest. **(F)** The Venn diagram shows the intersecting feature variables filtered by the three algorithms.

On the basis of the above analysis, we observed that, the higher the KIFscore, the worse the prognosis and that the KIFscore significantly affected genomic mutations as well as immune infiltration. We hypothesized that because of these associations, KIFscores could act as prognostic predictors. Therefore, we further analyzed and verified this in individual cancer types. We divided patients with ccRCC into high- and low-KIFscore groups by the median KIFscore and performed differential analysis (|log2FC| > 1; padj < 0.05). Next, we obtained 343 differential genes. After the genes were intersected with 38 KIF genes, we finally got 8 KIF genes (KIF14, KIF15, KIF18B, KIF20A, KIF23, KIF26A, KIF2C, and KIF4A). Next, we continued to screen hub-genes using different bioinformatics methods. Using the LASSO regression algorithm, we selected 5 genes as potentially pivotal genes (**Figures 7A, B**). The random forest (RF) algorithm ranked the 8 candidate genes row importance scores (**Figures 7C, D**). The SVM–RFE algorithm showed that when the number of characteristic genes was 5, the error was the lowest, at 0.191 (**Figure 7E**). Finally, we obtained the four genes, KIF26A, KIF20A, KIF23, and KIF14, as the key genes of ccRCC (**Figure 7F**).

### 3.7 The function of characteristic genes in clear cell renal cell carcinoma

**Figure 8:**
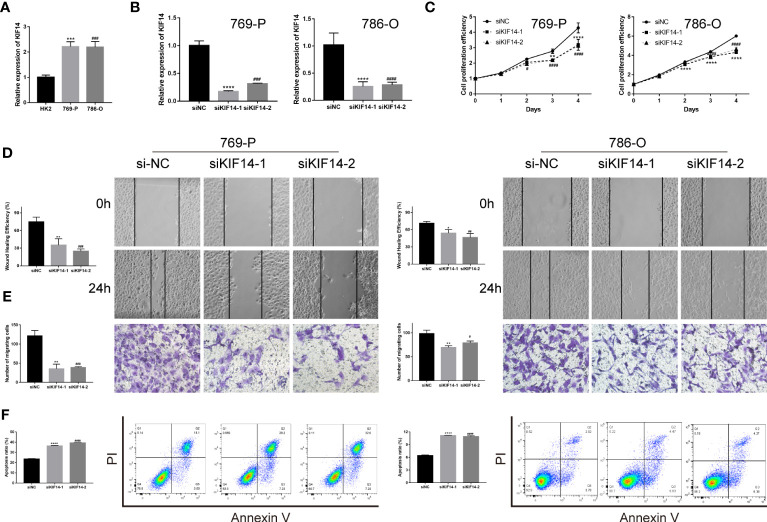
The functional verification of KIF14. **(A)** qRT−PCR verified the KIF14 expression in 769-P, 786-O, and HK2. **(B)** qRT−PCR confirmed that siKIF14 could knock down KIF14 in 769-P and 786-O. **(C)** A CCK-8 assay showed the effect of KIF14 knockdown on proliferation in 769-P and 786-O. **(D, E)** A wound healing assay **(D)** and a Transwell assay **(E)** revealed the effect of KIF14 knockdown on migration. **(F)** Flow cytometry revealed the effect of KIF14 knockdown on apoptosis in 769-P and 786-O (*p < 0.05, **p < 0.01, ***p < 0.001, ****p < 0.0001, ^#^p < 0.05, ^##^p < 0.01, ^###p^ < 0.001, and ^####^p < 0.0001).

Previous investigators have demonstrated that KIF20A (22) and KIF23 (23) are highly expressed in clear cell renal cell carcinoma and promote tumor progression, which is consistent with our analysis. Our analysis showed that there was low expression of KIF26A in ccRCC, which may lead to difficulties in validating the function of this gene. Therefore, in our validation experiments, we excluded the above three genes. As per our analysis, KIF14 was highly expressed in ccRCC and in patients with higher KIF14 expression levels, the tumor was in a more advanced stage and the prognosis was worse (**Supplementary Figures 4A, B**). PCR experiments confirmed that higher levels of KIF14 were expressed in clear cell renal cell carcinoma lines such as 769-P and 786-O than in renal normal cells such as HK2 (**Figures 8A, B**). The CCK8 assay showed a significant decrease in the proliferation efficiency of 769-P and 786-O after KIF14 was knocked out (**Figure 8C**). The wound healing assay and the Transwell assay confirmed that the migration ability of 769-P and 786-O decreased significantly after KIF14 was knocked down (**Figures 8D, E**). Flow cytometry revealed that the apoptosis of 769-P and 786-O increased after KIF14 was knocked down (**Figure 8F**). All of the above show that KIF14 was highly expressed in ccRCC and played the function of oncogenes, an important factor in poor prognosis.

## 4 Discussion

In many cancers, KIF molecules play a key role by changing the distribution of genetic material in cells, leading to an out-of-control cell cycle and progressively leading to tumor development. We developed a novel computational method (KIFscore) to analyze the role of KIF genes in various cancers. By investigating their correlation with clinical prognosis, genomic characteristics, and immunophenotyping, we identified specific associations in each cancer type. This knowledge can aid in selecting targeted therapies for KIFs in specific cancers. Furthermore, we identified four KIF genes as prognostic biomarkers, with experimental validation confirming the role of KIF14 in promoting tumor progression in ccRCC. Our computational metrics provide a framework for understanding the significance of KIF genes in tumors and can guide future experiments and biomarker identification.

Our investigation revealed that most KIF genes exhibit elevated basal expression levels in tumors compared to normal tissues. Additionally, these genes are associated with poor tumor prognosis, suggesting their potential role in tumor development. Notably, genes such as KIF11, KIF14, KIF15, KIF18A, KIF18B, KIF20A, KIF23, KIF2C, KIF4A, and KIFC1 were consistently highly expressed in malignant tumors, indicating their involvement in tumor regulation. Previous studies have reported the overexpression of KIF11, KIF15, and KIF18B in various malignancies, including gallbladder cancer, oral cancer, meningioma, pancreatic cancer, and osteosarcoma (24–28), supporting our findings.

We developed a novel method to calculate KIFscores, which allowed us to evaluate the expression levels and prognostic value of 38 KIF genes in tumors (29).. Our findings demonstrated that KIFscores were higher in tumor cells and tissues compared to normal tissues, and they served as risk factors for most tumors. Higher KIFscores correlated with worse prognosis, indicating their potential as prognostic indicators. Additionally, KIFscores showed associations with tumor grade and clinical indicators, reflecting their relevance to the degree of malignancy and clinical stage. These results align with the analysis of individual KIF genes, validating the reliability of KIFscores in measuring KIF gene levels in tumors and their prognostic evaluation value.

Genomic instability and mutation are fundamental components of cancer formation and pathogenesis (30). However, how genomic alterations vary with KIFscore in multiple cancers remains to be illustrated. To further explore the correlation between the KIFscore and genomic variations, we analyzed the correlation between the KIFscore and CNVs and found that the KIFscore was positively correlated with the CNV score of 21 malignant tumors. CNV gains and losses may activate oncogenes and inactivate tumor suppressor genes, leading to tumor development. In our research, the KIFscore was associated with additions on chromosomes 9q, 17p, and 20q and deletions on chromosomes 1q, 2q, 13q, and 16p. It was confirmed that the additions on 20q occurred in about 23% of ccRCC cases and was associated with resistance to four TKIs: Sunitinib, Cabozantinib, Axitinib, and Sorafenib. Furthermore, FoxO signaling was mechanistically associated with the addition on chromosome 20q (31). The deletion on chromosome 13q was an independent adverse risk factor for OS of ccRCC (32). The KIFscore demonstrated a positive correlation with tumor mutation burden (TMB) in most malignant tumors, indicating a significant relationship between KIFscores and genomic instability at the pan-cancer level. Substantial changes in KIFscores may play a crucial role in human cancer development. Specifically, in clear cell renal cell carcinoma (ccRCC), high and low KIFscores were associated with distinct gene mutation patterns that contribute to tumor progression. In the high-KIFscore group, VHL variations were primarily missense mutations, while in the low-KIFscore group, there was a notable increase in nonsense mutations. VHL mutations are known to activate oncogenes and promote ccRCC development (33). Additionally, patients with high KIFscores exhibited a higher proportion of poor prognostic immune subtypes, along with increased tumor stemness and DNA repair defects. These findings support the notion that ccRCC with high KIFscores tends to exhibit a higher degree of malignancy (34, 35).

The high-KIFscore group has a higher proportion of immune subtypes with poor prognosis. Further exploration of the correlation between the KIFscore and immune cell infiltration will help to explore its role in the immune microenvironment and explore the underlying mechanism. The high-KIFscore group has a higher proportion of immune subtypes with poor prognosis. Further exploration of the correlation between the KIFscore and immune cell infiltration will help to explore its role in the immune microenvironment and explore the underlying mechanism. In almost all cancers, the KIFscore was significantly positively correlated with T cell CD4+ TH2, the common lymphoid progenitor, and the T cell follicular helper. Normally, the Th1/Th2 ratio is in equilibrium. It was shown that cytokines released by Th2 cells and Th2 are elevated in various human cancers (36) and patients with a Th2-dominant response have a worse prognosis than patients with Th1/Th2 balance (37, 38). We also found that in LIHC, THCA, KIRP, and KIRC, the KIFscore was positively correlated with Treg cells. High levels of regulatory T cell (Treg) were found to be associated with reduced clinical benefit of hepatocellular carcinoma (39) and aggressive papillary thyroid carcinoma (40). In localized ccRCC, infiltration with ICOS+ Treg identifies patients with deleterious prognosis (41). As per this study and previous studies, high KIFscores could predict the poor prognosis of these malignant tumors. As per studies, CXCL16 could help to locally sustain the cytotoxic T lymphocyte (CTL) response for effective tumor control (42) and the overexpression of CCL14 could inhibit the proliferation of hepatoma cells and promote apoptosis (43). In most malignant tumors, the KIFscore was negatively correlated with CXCL16 and CCL14, suggesting that high KIFscores are not conducive to CTL responses against tumors. In most malignant tumors, the KIFscore was found to be positively correlated with ULBP1, MICB, and CD276, among which, CD276 expression was reported to enable squamous cell carcinoma stem cells to evade immune surveillance (44). Chen and Mellman proposed the concept of the cancer-immunity cycle, revealing the mechanism by which the immune system kills tumor cells (45). As per our analysis, the KIFscore influenced steps 1, 3, 4, and 6 of ccRCC and some critical steps in tumor immunity were more significant in the high-KIFscore group. These indicate that KIFs may play an important role in the tumor immune cycle.

KIFscores have significant implications for genomic mutations and immune infiltration, making them valuable prognostic predictors in tumors. By leveraging the multifaceted roles of KIFscores, we can identify promising candidate genes. Moreover, exploring meaningful KIF genes within a specific cancer type helps unveil potential therapeutic targets and areas for further investigation. Through machine learning, we identified four pivotal KIF genes in ccRCC, with KIF14 receiving extensive experimental validation. Its role in promoting proliferation and migration in renal clear cancer cells reaffirms the strong association between KIF family genes and tumor progression. Importantly, these findings highlight the potential of KIF14 as a novel therapeutic target for ccRCC.

## 5 Conclusion

In conclusion, we conducted a comprehensive pan-cancer analysis of KIF genes and introduced a novel computational metric called KIFscore. Our findings demonstrated that KIFscore effectively measures the expression levels of KIF genes and provides valuable insights into prognosis, genomic variations, and tumor immunity across multiple cancers. Through machine learning, we identified four key KIF genes in ccRCC, with KIF14 being highly expressed. Experimental validation showed that suppressing KIF14 significantly inhibited the proliferation and migration of ccRCC cells while promoting apoptosis. These results emphasize the broad significance of KIF genes in various cancers and suggest their potential as future therapeutic targets. Notably, KIF14 emerges as a crucial diagnostic and therapeutic marker for ccRCC.

## Data availability statement

The datasets presented in this study can be found in online repositories. The names of the repository/repositories and accession number(s) can be found in the article/**Supplementary Material**.

## Ethics statement

TCGA and GTEx belong to public databases. The patients involved in the database have obtained ethical approval. Users can download relevant data for free for research and publish relevant articles. Our study is based on open-source data, so there are no ethical issues and other conflicts of interest.

## Author contributions

MZ analyzed and interpreted the data. MZ, LG and NL wrote the manuscript. HG, KG, YZ, EZ, XW, SJ, JL, YaL, YuL and JC edited the manuscript. ZZ designed and edited the manuscript. All authors contributed to the article and approved the manuscript.

## References

[1] Yuan S Huang Z Qian X Wang Y Fang C Chen R. Pan-cancer analysis of the FAM83 family and its association with prognosis and tumor microenvironment. Front In Genet (2022) 13:919559. doi: 10.3389/fgene.2022.919559 35938024 PMC9353330

[2] Song J Tang Y Luo X Shi X Song F Ran L. Pan-cancer analysis reveals the signature of TMC family of genes as a promising biomarker for prognosis and immunotherapeutic response. Front In Immunol (2021) 12:715508. doi: 10.3389/fimmu.2021.715508 34899684 PMC8660091

[3] Lucanus AJ Yip GW. Kinesin superfamily: roles in breast cancer, patient prognosis and therapeutics. Oncogene (2018) 37(7):833–8. doi: 10.1038/onc.2017.406 29059174

[4] Rath O Kozielski F. Kinesins and cancer. Nat Rev Cancer (2012) 12(8):527–39. doi: 10.1038/nrc3310 22825217

[5] Hirokawa N Noda Y Tanaka Y Niwa S. Kinesin superfamily motor proteins and intracellular transport. Nat Rev Mol Cell Biol (2009) 10(10):682–96. doi: 10.1038/nrm2774 19773780

[6] Gifford V Woskowicz A Ito N Balint S Lagerholm BC Dustin ML. Coordination of two kinesin superfamily motor proteins, KIF3A and KIF13A, is essential for pericellular matrix degradation by membrane-type 1 matrix metalloproteinase (MT1-MMP) in cancer cells. Matrix Biol (2022) 107:1–23. doi: 10.1016/j.matbio.2022.01.004 35122963 PMC9355896

[7] Eichenlaub-Ritter U. Microtubule dynamics and tumor invasion involving MCAK. Cell Cycle (2015) 14(21):3353. doi: 10.1080/15384101.2015.1093813 26375511 PMC4825562

[8] Ganguly A Yang H Pedroza M Bhattacharya R Cabral F. Mitotic centromere-associated kinesin (MCAK) mediates paclitaxel resistance. J Biol Chem (2011) 286(42):36378–84. doi: 10.1074/jbc.M111.296483 PMC3196137 21903575

[9] Gao L Zhang W Zhang J Liu J Sun F Liu H. KIF15-mediated stabilization of AR and AR-V7 contributes to enzalutamide resistance in prostate cancer. Cancer Res (2021) 81(4):1026–39. doi: 10.1158/0008-5472.CAN-20-1965 33277366

[10] Copello VA Burnstein KL. The kinesin KIF20A promotes progression to castration-resistant prostate cancer through autocrine activation of the androgen receptor. Oncogene (2022) 41(20):2824–32. doi: 10.1038/s41388-022-02307-9 PMC9107495 35418689

[11] Colaprico A Silva TC Olsen C Garofano L Cava C Garolini D. TCGAbiolinks: an R/Bioconductor package for integrative analysis of TCGA data. Nucleic Acids Res (2016) 44(8):e71. doi: 10.1093/nar/gkv1507 26704973 PMC4856967

[12] Hänzelmann S Castelo R Guinney J. GSVA: gene set variation analysis for microarray and RNA-seq data. BMC Bioinf (2013) 14:7. doi: 10.1186/1471-2105-14-7 PMC3618321 23323831

[13] Zhong M Wang X Zhu E Gong L Fei L Zhao L. Analysis of pyroptosis-related immune signatures and identification of pyroptosis-related lncRNA prognostic signature in clear cell renal cell carcinoma. Front In Genet (2022) 13:905051. doi: 10.3389/fgene.2022.905051 35846134 PMC9277062

[14] Gong L Zhong M Gong K Wang Z Zhong Y Jin Y. Multi-omics analysis and verification of the oncogenic value of CCT8 in pan-cancers. J Inflammation Res (2023) 16:2297–315. doi: 10.2147/JIR.S403499 PMC10238552 37273485

[15] Mermel CH Schumacher SE Hill B Meyerson ML Beroukhim R Getz G. GISTIC2.0 facilitates sensitive and confident localization of the targets of focal somatic copy-number alteration in human cancers. Genome Biol (2011) 12(4):R41. doi: 10.1186/gb-2011-12-4-r41 21527027 PMC3218867

[16] Yuan J Hu Z Mahal BA Zhao SD Kensler KH Pi J. Integrated analysis of genetic ancestry and genomic alterations across cancers. Cancer Cell (2018) 34(4):549–60. doi: 10.1016/j.ccell.2018.08.019 PMC6348897 30300578

[17] Mayakonda A Lin D-C Assenov Y Plass C Koeffler HP. Maftools: efficient and comprehensive analysis of somatic variants in cancer. Genome Res (2018) 28(11):1747–56. doi: 10.1101/gr.239244.118 PMC6211645 30341162

[18] Engebretsen S Bohlin J. Statistical predictions with glmnet. Clin Epigenet (2019) 11(1):123. doi: 10.1186/s13148-019-0730-1 PMC6708235 31443682

[19] Sanz H Valim C Vegas E Oller JM Reverter F. SVM-RFE: selection and visualization of the most relevant features through non-linear kernels. BMC Bioinf (2018) 19(1):432. doi: 10.1186/s12859-018-2451-4 PMC6245920 30453885

[20] Zhong M Zhu E Li N Gong L Xu H Zhong Y. Identification of diagnostic markers related to oxidative stress and inflammatory response in diabetic kidney disease by machine learning algorithms: Evidence from human transcriptomic data and mouse experiments. Front In Endocrinol (2023) 14:1134325. doi: 10.3389/fendo.2023.1134325 PMC10028207 36960398

[21] Tang T Zhu Z He Z Wang F Chen H Liu S. DLX5 regulates the osteogenic differentiation of spinal ligaments cells derived from ossification of the posterior longitudinal ligament patients *via* NOTCH signaling. JOR Spine (2023) 6(2):e1247. doi: 10.1002/jsp2.1247 37361333 PMC10285757

[22] Ren X Chen X Ji Y Li L Li Y Qin C. Upregulation of KIF20A promotes tumor proliferation and invasion in renal clear cell carcinoma and is associated with adverse clinical outcome. Aging (2020) 12(24):25878–94. doi: 10.18632/aging.202153 PMC7803492 33232285

[23] Wu Z Song Y Wu Y Ge L Liu Z Du T. Identification of KIF23 as a prognostic biomarker associated with progression of clear cell renal cell carcinoma. Front In Cell Dev Biol (2022) 10:839821. doi: 10.3389/fcell.2022.839821 35478956 PMC9035542

[24] Daigo K Takano A Thang PM Yoshitake Y Shinohara M Tohnai I. Characterization of KIF11 as a novel prognostic biomarker and therapeutic target for oral cancer. Int J Oncol (2018) 52(1):155–65. doi: 10.3892/ijo.2017.4181 PMC5743338 29115586

[25] Gao T Yu L Fang Z Liu J Bai C Li S. KIF18B promotes tumor progression in osteosarcoma by activating β-catenin. Cancer Biol Med (2020) 17(2):371–86. doi: 10.20892/j.issn.2095-3941.2019.0452 PMC7309474 32587775

[26] Jungwirth G Yu T Moustafa M Rapp C Warta R Jungk C. Identification of KIF11 as a novel target in meningioma. Cancers (Basel) (2019) 11(4):545. doi: 10.3390/cancers11040545 30991738 PMC6521001

[27] Mi J Ma S Chen W Kang M Xu M Liu C. Integrative pan-cancer analysis of KIF15 reveals its diagnosis and prognosis value in nasopharyngeal carcinoma. Front In Oncol (2022) 12:772816. doi: 10.3389/fonc.2022.772816 PMC8963360 35359374

[28] Wei D Rui B Qingquan F Chen C Ping HY Xiaoling S. KIF11 promotes cell proliferation *via* ERBB2/PI3K/AKT signaling pathway in gallbladder cancer. Int J Biol Sci (2021) 17(2):514–26. doi: 10.7150/ijbs.54074 PMC7893577 33613109

[29] Wang X Ma L Pei X Wang H Tang X Pei J-F. Comprehensive assessment of cellular senescence in the tumor microenvironment. Brief Bioinform (2022) 23(3):bbac118. doi: 10.1093/bib/bbac118 35419596 PMC9116224

[30] Hanahan D Weinberg RA. Hallmarks of cancer: the next generation. Cell (2011) 144(5):646–74. doi: 10.1016/j.cell.2011.02.013 21376230

[31] Wang L Li Y Lyu Y Wen H Feng C. Association between copy-number alteration of +20q, -14q and -18p and cross-sensitivity to tyrosine kinase inhibitors in clear-cell renal cell carcinoma. Cancer Cell Int (2020) 20:482. doi: 10.1186/s12935-020-01585-1 33041663 PMC7541266

[32] Xiong Y Qi Y Bai Q Xia Y Liu L Guo J. Relevance of arm somatic copy number alterations for oncologic outcomes and tumor immune microenvironment in clear cell renal cell carcinoma. Ann Transl Med (2019) 7(22):646. doi: 10.21037/atm.2019.10.54 31930047 PMC6944545

[33] Stone L. Kidney cancer: Activation of oncogenes driven by VHL loss in ccRCC. Nat Rev Urol (2017) 14(11):637. doi: 10.1038/nrurol.2017.162 28976497

[34] Lytle NK Barber AG Reya T. Stem cell fate in cancer growth, progression and therapy resistance. Nat Rev Cancer (2018) 18(11):669–80. doi: 10.1038/s41568-018-0056-x PMC8388042 30228301

[35] Pattabiraman DR Weinberg RA. Tackling the cancer stem cells - what challenges do they pose? Nat Rev Drug Discovery (2014) 13(7):497–512. doi: 10.1038/nrd4253 24981363 PMC4234172

[36] Saravia J Chapman NM Chi H. Helper T cell differentiation. Cell Mol Immunol (2019) 16(7):634–43. doi: 10.1038/s41423-019-0220-6 PMC6804569 30867582

[37] DeNardo DG Barreto JB Andreu P Vasquez L Tawfik D Kolhatkar N. CD4(+) T cells regulate pulmonary metastasis of mammary carcinomas by enhancing protumor properties of macrophages. Cancer Cell (2009) 16(2):91–102. doi: 10.1016/j.ccr.2009.06.018 19647220 PMC2778576

[38] Qin H Lu Y Du L Shi J Yin H Jiang B. Pan-cancer analysis identifies LMNB1 as a target to redress Th1/Th2 imbalance and enhance PARP inhibitor response in human cancers. Cancer Cell Int (2022) 22(1):101. doi: 10.1186/s12935-022-02467-4 35241075 PMC8896121

[39] Zhu AX Abbas AR de Galarreta MR Guan Y Lu S Koeppen H. Molecular correlates of clinical response and resistance to atezolizumab in combination with bevacizumab in advanced hepatocellular carcinoma. Nat Med (2022) 28(8):1599–611. doi: 10.1038/s41591-022-01868-2 35739268

[40] Liu Y Yun X Gao M Yu Y Li X. Analysis of regulatory T cells frequency in peripheral blood and tumor tissues in papillary thyroid carcinoma with and without Hashimoto’s thyroiditis. Clin Transl Oncol (2015) 17(4):274–80. doi: 10.1007/s12094-014-1222-6 25387566

[41] Giraldo NA Becht E Vano Y Petitprez F Lacroix L Validire P. Tumor-infiltrating and peripheral blood T-cell immunophenotypes predict early relapse in localized clear cell renal cell carcinoma. Clin Cancer Res (2017) 23(15):4416–28. doi: 10.1158/1078-0432.CCR-16-2848 28213366

[42] Di Pilato M Kfuri-Rubens R Pruessmann JN Ozga AJ Messemaker M Cadilha BL. CXCR6 positions cytotoxic T cells to receive critical survival signals in the tumor microenvironment. Cell (2021) 184(17):4512–30. doi: 10.1016/j.cell.2021.07.015 PMC8719451 34343496

[43] Zhu M Xu W Wei C Huang J Xu J Zhang Y. CCL14 serves as a novel prognostic factor and tumor suppressor of HCC by modulating cell cycle and promoting apoptosis. Cell Death Dis (2019) 10(11):796. doi: 10.1038/s41419-019-1966-6 31641099 PMC6805940

[44] Wang C Li Y Jia L Kim JK Li J Deng P. CD276 expression enables squamous cell carcinoma stem cells to evade immune surveillance. Cell Stem Cell (2021) 28(9):1597–613. doi: 10.1016/j.stem.2021.04.011 PMC8419062 33945793

[45] Chen DS Mellman I. Oncology meets immunology: the cancer-immunity cycle. Immunity (2013) 39(1);1–10. doi: 10.1016/j.immuni.2013.07.012 23890059

