# Supplementary material for: Pan-cancer analysis of kinesin family members with potential implications in prognosis and immunological role in human cancer
Source: Front Oncol. 2023 Aug 29;13:1179897. doi: 10.3389/fonc.2023.1179897 (PMC10498125; doi:10.3389/fonc.2023.1179897)
Supplement: Supplementary file 1 [file DataSheet_1.docx]

**Supplementary Figures**

**Supplementary Figure 1.**

**
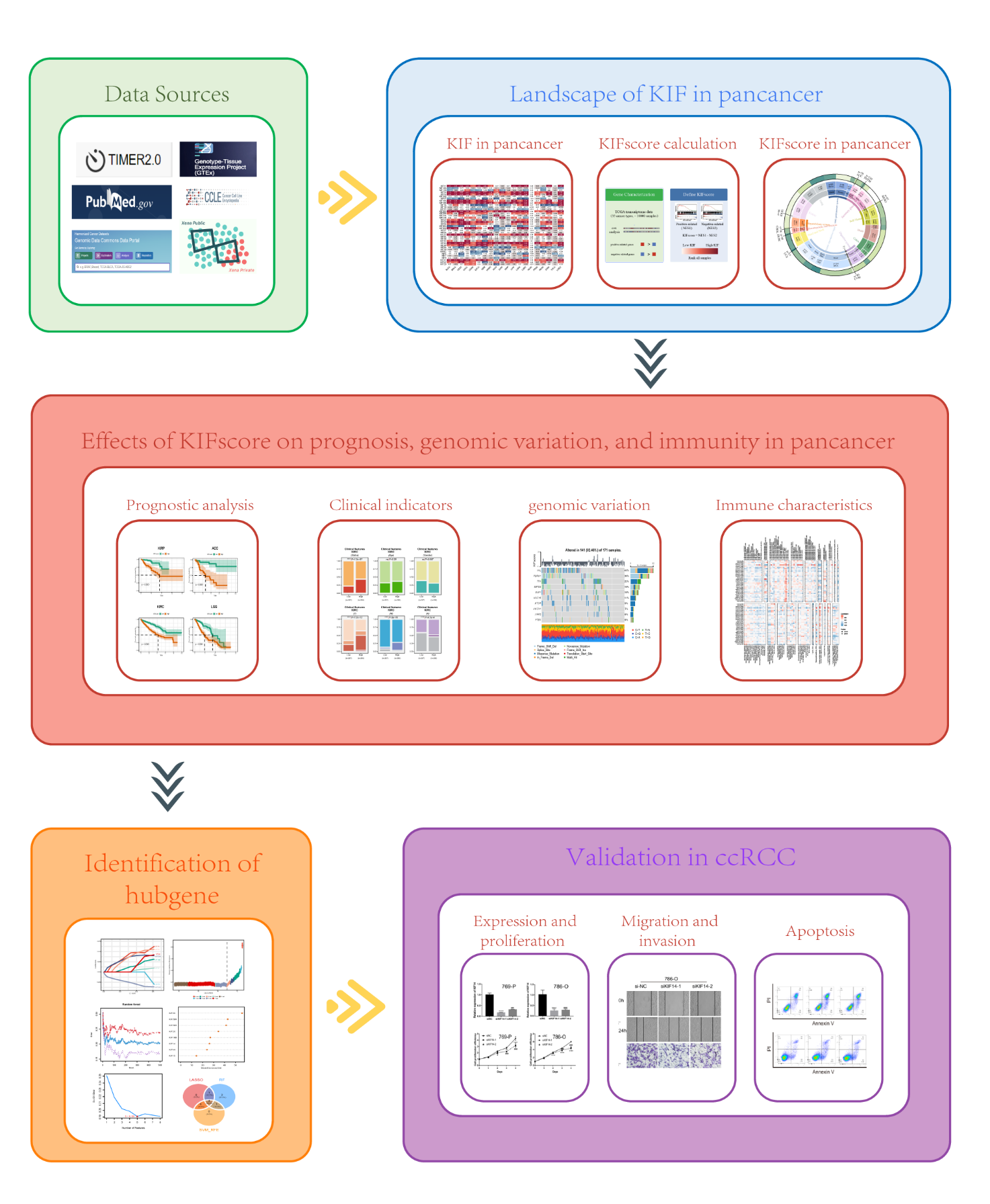
**

**Supplementary Figure 1.** Workflow for this study. The text in the diagram described the main processes of each stage.

**Supplementary Figure 2.**

**
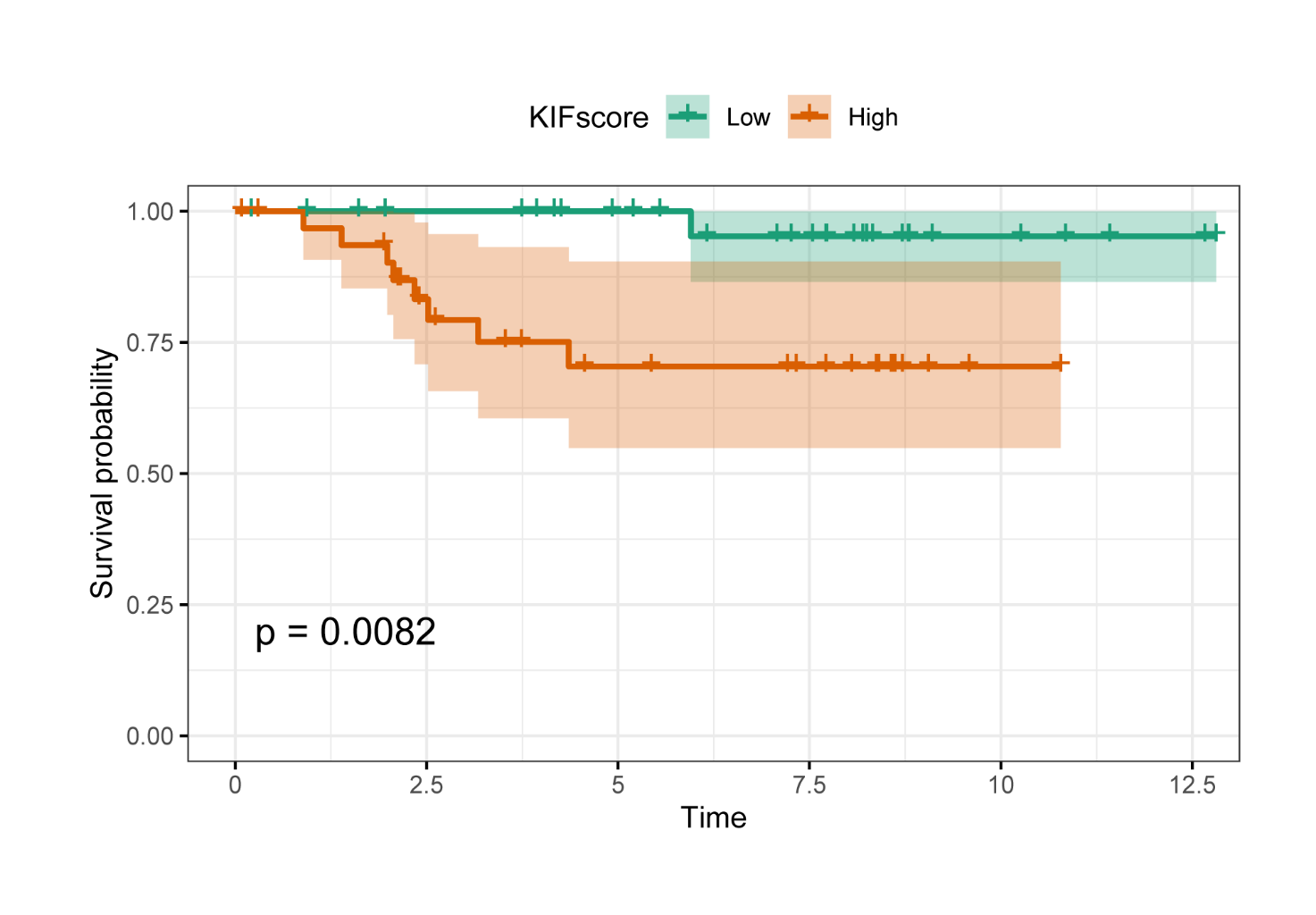
**

**Supplementary Figure 2.** Survival analysis curve for Kidney Chromophobe (KICH).

**Supplementary Figure 3.**

**
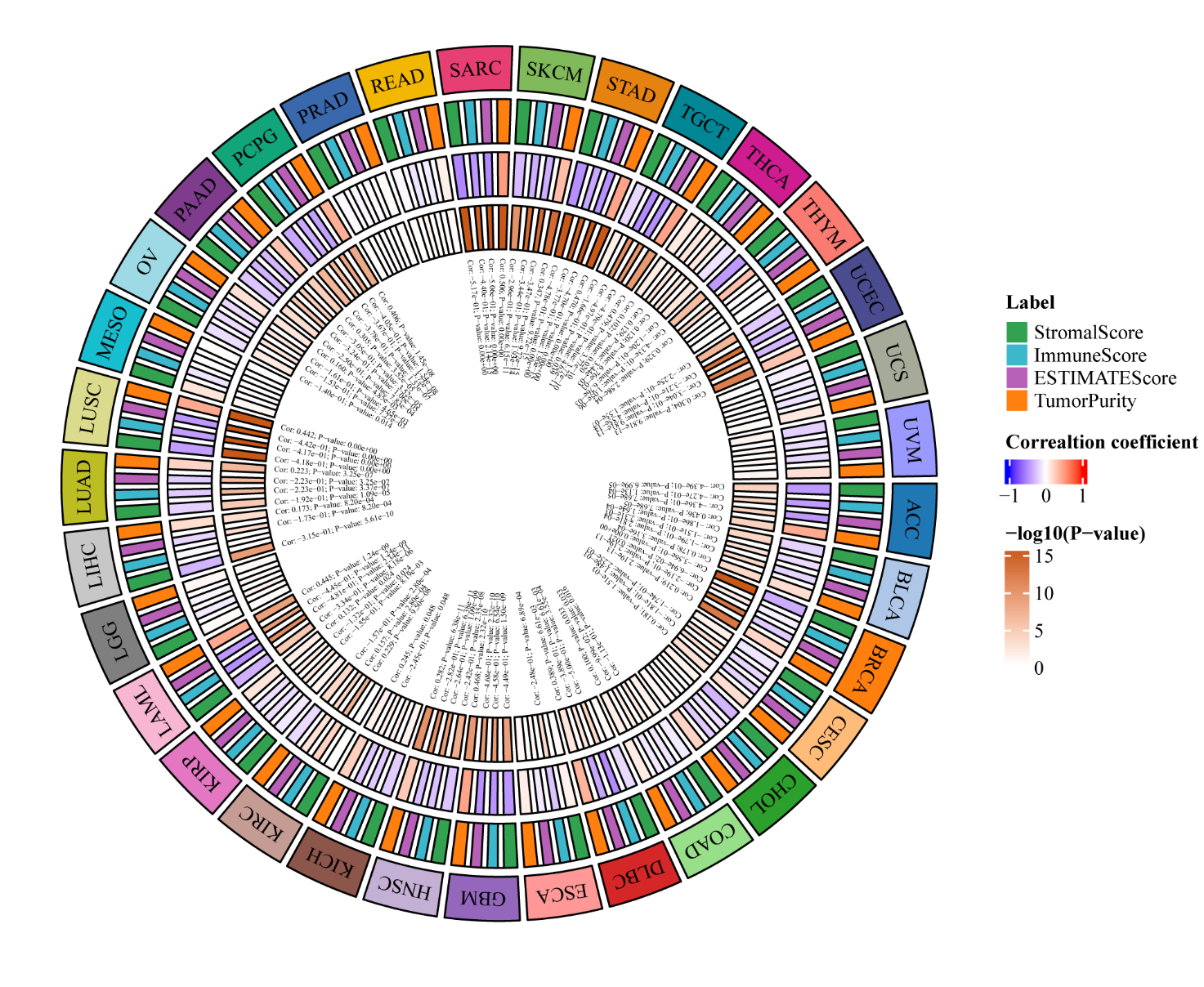
**

**Supplementary Figure 3.** Correlation between KIFscore and tumor microenvironment score in pan-cancer. From outside the circle to inside were the tumor type, tumor microenvironment score, correlation coefficient and P value, respectively.

**Supplementary Figure 4.**

**
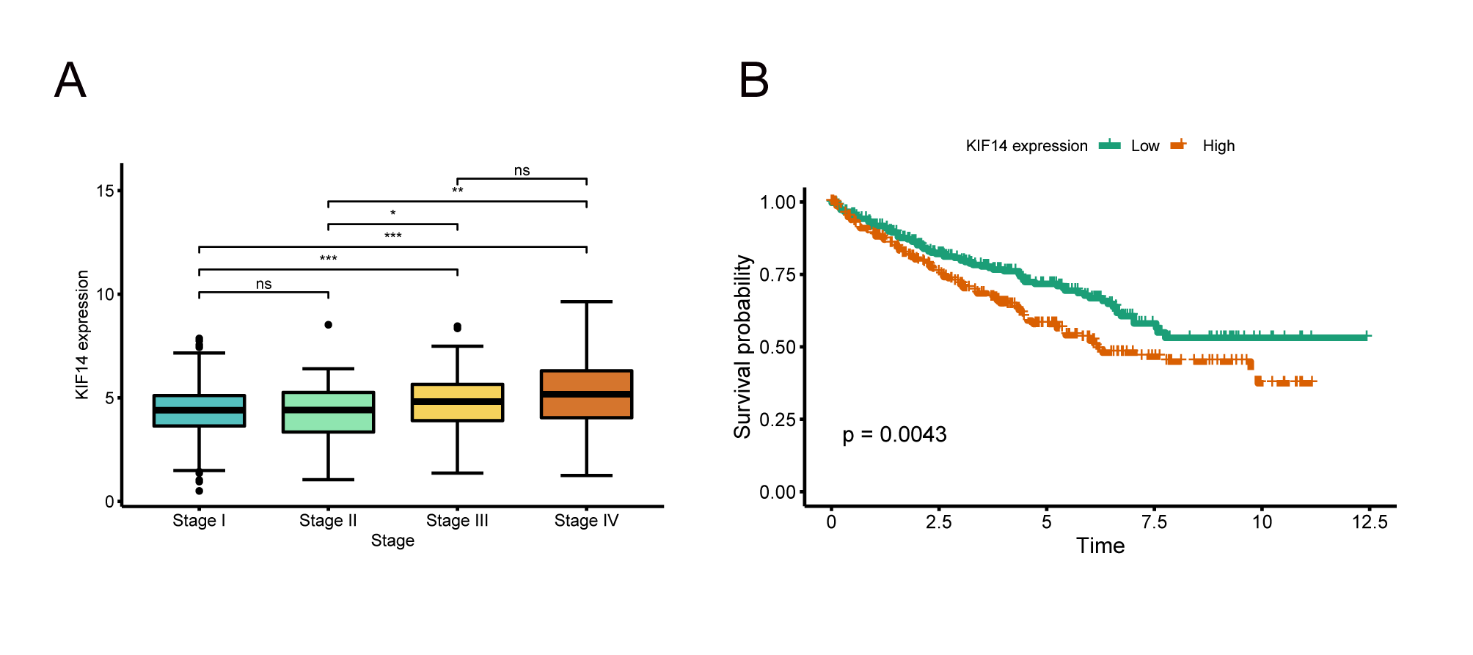
**

**Supplementary Figure 4.** Correlation between KIF14 and clinical staging and prognosis of ccRCC. (A) The boxplot shown the expression of KIF14 in patients with ccRCC at different stages. (B) Effect of the expression level of KIF14 on the prognosis of ccRCC.
